# Metabolic crosstalk between hydroxylated monoterpenes and salicylic acid in tomato defense response against bacteria

**DOI:** 10.1093/plphys/kiae148

**Published:** 2024-03-13

**Authors:** Julia Pérez-Pérez, Samuel Minguillón, Elías Kabbas-Piñango, Celia Payá, Laura Campos, Manuel Rodríguez-Concepción, Ana Espinosa-Ruiz, Ismael Rodrigo, José María Bellés, María Pilar López-Gresa, Purificación Lisón

**Affiliations:** Instituto de Biología Molecular y Celular de Plantas (IBMCP), Consejo Superior de Investigaciones Científicas (CSIC), Universitat Politècnica de València (UPV), Ciudad Politécnica de la Innovación (CPI) 8 E, Ingeniero Fausto Elio s/n, 46011 Valencia, Spain; Instituto de Biología Molecular y Celular de Plantas (IBMCP), Consejo Superior de Investigaciones Científicas (CSIC), Universitat Politècnica de València (UPV), Ciudad Politécnica de la Innovación (CPI) 8 E, Ingeniero Fausto Elio s/n, 46011 Valencia, Spain; Instituto de Biología Molecular y Celular de Plantas (IBMCP), Consejo Superior de Investigaciones Científicas (CSIC), Universitat Politècnica de València (UPV), Ciudad Politécnica de la Innovación (CPI) 8 E, Ingeniero Fausto Elio s/n, 46011 Valencia, Spain; Instituto de Biología Molecular y Celular de Plantas (IBMCP), Consejo Superior de Investigaciones Científicas (CSIC), Universitat Politècnica de València (UPV), Ciudad Politécnica de la Innovación (CPI) 8 E, Ingeniero Fausto Elio s/n, 46011 Valencia, Spain; Instituto de Biología Molecular y Celular de Plantas (IBMCP), Consejo Superior de Investigaciones Científicas (CSIC), Universitat Politècnica de València (UPV), Ciudad Politécnica de la Innovación (CPI) 8 E, Ingeniero Fausto Elio s/n, 46011 Valencia, Spain; Instituto de Biología Molecular y Celular de Plantas (IBMCP), Consejo Superior de Investigaciones Científicas (CSIC), Universitat Politècnica de València (UPV), Ciudad Politécnica de la Innovación (CPI) 8 E, Ingeniero Fausto Elio s/n, 46011 Valencia, Spain; Instituto de Biología Molecular y Celular de Plantas (IBMCP), Consejo Superior de Investigaciones Científicas (CSIC), Universitat Politècnica de València (UPV), Ciudad Politécnica de la Innovación (CPI) 8 E, Ingeniero Fausto Elio s/n, 46011 Valencia, Spain; Instituto de Biología Molecular y Celular de Plantas (IBMCP), Consejo Superior de Investigaciones Científicas (CSIC), Universitat Politècnica de València (UPV), Ciudad Politécnica de la Innovación (CPI) 8 E, Ingeniero Fausto Elio s/n, 46011 Valencia, Spain; Instituto de Biología Molecular y Celular de Plantas (IBMCP), Consejo Superior de Investigaciones Científicas (CSIC), Universitat Politècnica de València (UPV), Ciudad Politécnica de la Innovación (CPI) 8 E, Ingeniero Fausto Elio s/n, 46011 Valencia, Spain; Instituto de Biología Molecular y Celular de Plantas (IBMCP), Consejo Superior de Investigaciones Científicas (CSIC), Universitat Politècnica de València (UPV), Ciudad Politécnica de la Innovación (CPI) 8 E, Ingeniero Fausto Elio s/n, 46011 Valencia, Spain; Instituto de Biología Molecular y Celular de Plantas (IBMCP), Consejo Superior de Investigaciones Científicas (CSIC), Universitat Politècnica de València (UPV), Ciudad Politécnica de la Innovación (CPI) 8 E, Ingeniero Fausto Elio s/n, 46011 Valencia, Spain

## Abstract

Hydroxylated monoterpenes (HMTPs) are differentially emitted by tomato (*Solanum lycopersicum*) plants resisting bacterial infection. We have studied the defensive role of these volatiles in the tomato response to bacteria, whose main entrance is through stomatal apertures. Treatments with some HMTPs resulted in stomatal closure and pathogenesis-related protein 1 (*PR1*) induction. Particularly, α-terpineol induced stomatal closure in a salicylic acid (SA) and abscisic acid-independent manner and conferred resistance to bacteria. Interestingly, transgenic tomato plants overexpressing or silencing the monoterpene synthase *MTS1*, which displayed alterations in the emission of HMTPs, exhibited changes in the stomatal aperture but not in plant resistance. Measures of both 2-*C*-methyl-D-erythritol-2,4-cyclopyrophosphate (MEcPP) and SA levels revealed competition for MEcPP by the methylerythritol phosphate (MEP) pathway and SA biosynthesis activation, thus explaining the absence of resistance in transgenic plants. These results were confirmed by chemical inhibition of the MEP pathway, which alters MEcPP levels. Treatments with benzothiadiazole (BTH), a SA functional analog, conferred enhanced resistance to transgenic tomato plants overexpressing *MTS1*. Additionally, these *MTS1* overexpressors induced *PR1* gene expression and stomatal closure in neighboring plants. Our results confirm the role of HMTPs in both intra- and interplant immune signaling and reveal a metabolic crosstalk between the MEP and SA pathways in tomato plants.

## Teaser

Hydroxylated monoterpenes play an important role in both intra- and interplant immune signaling in tomato against bacterial infection.

## Introduction

Understanding the defense signaling pathways in plants has led to the discovery of resistance-inducing compounds for the agrochemical sector (López-Gresa et al. 2018). These compounds may act directly as powerful antioxidants, antibacterial, or antifungal agents against the pathogen or act indirectly by activating the plant defense response. In addition to alkaloids and phenolics, some volatile organic compounds (VOCs) belong to this group of defensive molecules (Junker and Tholl 2013; Brosset and Blande 2022).

One of the most diverse types of VOCs is terpenoids, also known as isoprenoids, and includes a very extensive and varied set of molecules. They are formed from repeating units of 5-carbon (C5) isoprene building blocks—named isopentenyl diphosphate and dimethylallyl diphosphate—which are obtained from mevalonic acid (MVA) or methylerythritol phosphate (MEP) in the cytosol or plastids, respectively (Degenhardt et al. 2009). Particularly, monoterpenes consist of 2 isoprene units (C10) and can be modified with different functional groups, such as the addition of a hydroxyl to form the hydroxylated monoterpenes (HMTPs). Terpene synthases (TPSs) catalyze the synthesis of isoprenoids, being responsible for the diversity of terpenoids found in nature (Tholl and Lee 2011; Karunanithi and Zerbe 2019). The analysis of the updated tomato (*Solanum lycopersicum*) genome (2017 version of v. SL3.0) has revealed that there are 34 full-length *TPS* genes and 18 pseudo *TPS* genes. The biochemical analysis has identified the catalytic activities of all the enzymes encoded by all 34 *TPS* genes: an isoprene (C5) synthase, 10 exclusively or predominantly monoterpene (C10) synthases, 17 sesquiterpene (C15) synthases, and 6 diterpene (C20) synthases (Zhou and Pichersky 2020). Among TPSs, the recombinant protein of the monoterpene synthase MTS1 produces the monoterpenoid β-linalool but also the sesquiterpenoid β-nerolidol, generating its overexpression producing enhanced levels of linalool in tomato plants. This gene is induced by insects, wounding, and jasmonic acid (JA) treatment (Van Schie et al. 2007). Therefore, the defensive role of monoterpenes has been classically associated with plant–herbivore interaction, although the interest on its role in plant defense against pathogens is increasing (Vlot et al. 2021).

A nontargeted metabolomic analysis was performed using GC–MS to identify VOCs differentially emitted by Rio Grande (RG) tomato plants carrying the resistance to *Pseudomonas syringae* pathovar (pv.) *tomato Pto* gene, infected by an avirulent strain of *P. syringae* pv. *tomato*. The analysis of the specific volatilome from these plants, displaying the so-called effector-triggered immunity (ETI; Jones and Dangl 2006), revealed that the aroma characteristic of resistance includes (*Z*)-3-hexenol esters—including (*Z*)-3-hexenyl butanoate (HB)—as well as some HMTPs, such as α-terpineol, 4-terpineol, and linalool (López-Gresa et al. 2017). The defensive role of HB has already been demonstrated, since it produces stomatal closure and induces the defensive response, thus preventing the entry of bacteria (López-Gresa et al. 2018). This compound was patented (Lisón et al. 2018) for its potential uses in agriculture against biotic and abiotic stresses (Payá et al. 2020, 2024). However, the role of HMTPs in tomato immunity still remains unknown. In this respect, it is relevant to note that HMTPs, and particularly linalool, are synthesized through the MEP plastid pathway by the action of *MTS1*, which has been described to be induced during ETI in tomato plants (López-Gresa et al. 2017).

The defense response upon avirulent *P. syringae* pv. *tomato* infection also includes the activation of salicylic acid (SA) biosynthesis (Robert-Seilaniantz et al. 2011). This phytohormone is involved in different physiological and biochemical processes, and it is well characterized as a signaling molecule for the induction of different pathways that enhance in plant resistance (Klessig et al. 2018). This phenolic compound is biosynthesized in plants from phenylalanine through the route of the phenylpropanoids (PAL pathway) or from the isochorismate (IC pathway). Loss of function of some genes from both pathways results in an increased plant susceptibility to pathogens. Nevertheless, isochorismate synthase (ICS1) is the main enzyme of SA biosynthesis in biotic responses and produces 90% of its levels under biotic stress (Wildermuth et al. 2001). In plants, IC is conjugated to the amino acid L-glutamate to produce isochorismoyl-9-glutamate (IC-9-Glu) that can spontaneously break down into SA. Besides, EPS1 (ENHANCED PSEUDOMONAS SUSCEPTIBILITY 1), an IC-9-Glu pyruvoyl-glutamate lyase, enhances this process more effectively (Zeier 2021). To avoid the toxic effects caused by its accumulation, SA is chemically modified into different derivatives, through glycosylation, methylation, sulfonation, amino acid conjugation, and hydroxylation. Particularly, most of the SA present in the plant is glycosylated into SA 2-*O*-β-D-glucoside (SAG). In addition, SA can be methylated to form the volatile methyl salicylate (MeSA) or hydroxylated to form gentisic acid (GA) by the action of S5H (salicylic acid 5-hydroxylase; Bellés et al. 2006; Ding and Ding 2020; Payá et al. 2022).

The differential emission of HMTPs in an avirulent bacterial infection, as well as the observed induction of the monoterpene synthase *MTS1* (López-Gresa et al. 2017), prompted us to delve into the possible defensive role of HMTPs in tomato plants. In this context, the general objective of this work is to study the defensive role and the mode of action of HMTPs, including the possible interrelation with SA, in the tomato–bacteria interaction.

## Results

### HMTPs activate the plant defense response

**Figure 1.**
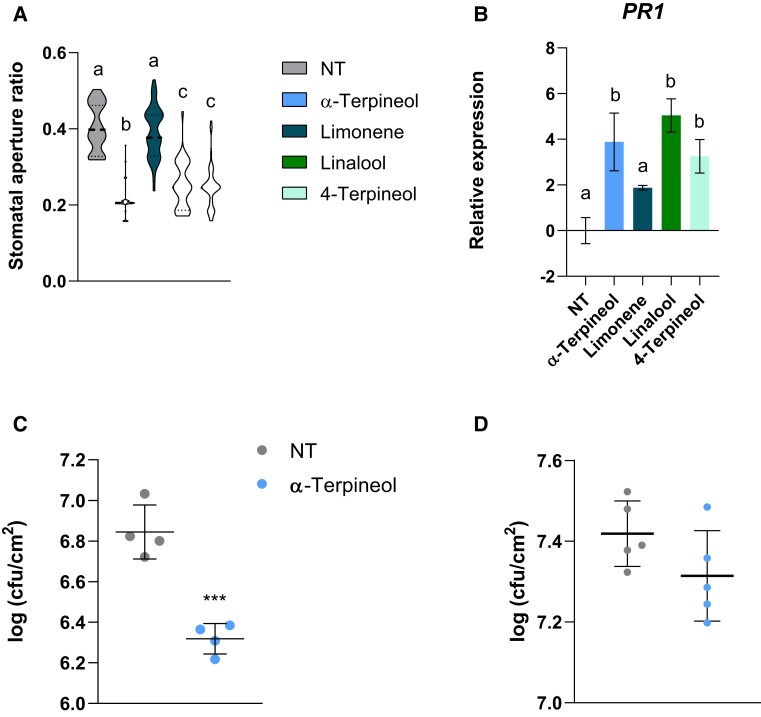
Effect of monoterpenoid treatments on the defensive response of MM tomato plants. **A)** Stomatal aperture ratio of nontreated (NT) tomato plants or treated with α-terpineol, limonene, linalool, and 4-terpineol. Violin plots represent the stomatal aperture ratio for each treatment of a total of 40 stomata from 3 biological replicates. Different letters indicate statistically significant differences for each treatment (ANOVA, *P* < 0.05). **B)** RT-qPCR analysis of the tomato *PR1* gene expression in NT tomato plants or treated with α-terpineol, limonene, linalool, and 4-terpineol. The *y* axis represents the value of the Ct increment (ΔΔCt). Values were normalized to Actin gene. Expression levels are represented as mean ± Sd of 3 biological replicates of 1 representative experiment. Letters represent statistically significant differences (ANOVA, *P* < 0.05) between treatments. Bacterial content 24 h after infection in tomato plants pretreated with 5 *µ*M α-terpineol or NT and then inoculated by **C)** immersion or **D)** injection 1 d later. Data are presented as mean (log cfu/cm^2^) ± Sd of a representative experiment (*n* = 4; *n* = 5). Statistically significant differences (*t* test, *P* < 0.001) between treated and NT plants are represented by triple asterisks in **C)**, and no statistically significant differences between treated and NT plants were observed in **D)**.

Stomata play a critical role in restricting bacterial invasion as part of the plant immune system (Underwood et al. 2007). In addition to biotic stress, stomata also respond to several volatile compounds, some of them with defensive activity (López-Gresa et al. 2018). In order to explore the defensive role of HMTPs whose differential emission is triggered by ETI, MoneyMaker (MM) tomato plants were treated with 5 *µ*M α-terpineol, 4-terpineol, or linalool, and both stomata closure and expression of pathogenesis-related protein 1 (*PR1*), the main marker gene of SA-mediated plant response to biotrophic attack (Tornero et al. 1997), were analyzed. In addition, treatments with the non-HMTP limonene were also performed. As observed in Fig. 1A, a significant stomatal closure occurred in α-terpineol, 4-terpineol, and linalool but not in limonene-treated plants. In a similar manner, HMTP treatments also produced a significant induction of *PR1* expression, while limonene had no significant effect (Fig. 1B). Our results appear to indicate that both stomatal closure and activation of *PR1* are specifically triggered by the hydroxylated forms of monoterpenes, being α-terpineol selected for further studies, since it was the compound producing the highest stomatal closure.

To confirm the defensive role of α-terpineol, MM tomato plants were pretreated with 5 *µ*M α-terpineol. This concentration was chosen according to the range of natural emission of HMTPs in wild type infected tomato plants. Then, pretreated tomato plants were subjected to *P. syringae* pv. *tomato* DC3000 (*Pst*) infection by immersion 1 d later (see Materials and methods). A significant induction of resistance was observed in α-terpineol-treated tomato plants after 24 h of bacterial inoculation, when compared with the nontreated plants (Fig. 1C). The efficacy of the pretreatment was also tested in the experiment, confirming the activation of PR1 by *Western blot* analysis (Supplementary Fig. S1).

To assess if the HMTP-mediated resistance was due to stomatal closure or *PR1* induction, α-terpineol-pretreated plants were infected by bacterial infiltration (see Materials and methods). Through this infection method, bacteria bypass the stomatal immunity and the *PR1* effect on plant defense could be independently evaluated. As Fig. 1D shows, resistance produced by α-terpineol lost statistical significance when bacteria were directly infiltrated in the plant, although a tendency to reduce the bacteria content was observed. These results indicate that the effect of α-terpineol on plant resistance is partially due to its capability to close the stomata.

### α-Terpineol triggers stomatal closure in an SA/ABA-independent manner and SA-dependent plant resistance

**Figure 2.**
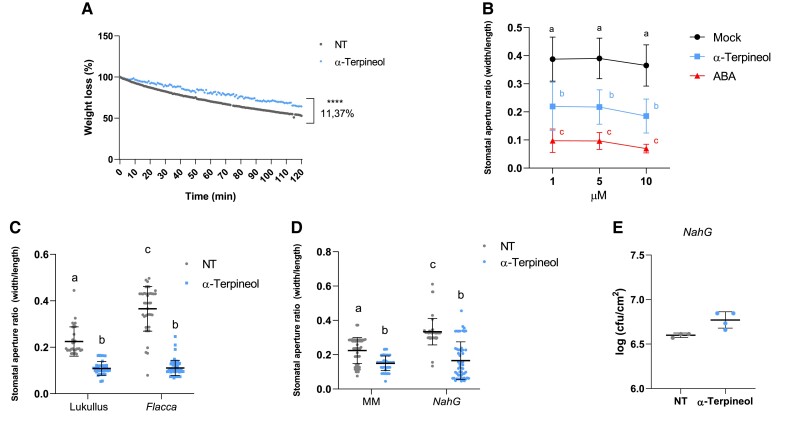
HMTP mode of action. **A)** Weight loss of nontreated (NT) or α-terpineol-treated (α-terpineol) tomato seedlings at different time points during 3 h. The experiment was repeated 3 times obtaining similar values, and 25 seedlings were used as described in Materials and methods (*t* test, *P* < 0.0001). **B)** Dose–response analysis of α-terpineol (blue), ABA (red), and mock (water, black) in stomatal aperture. Data represent the mean ± Sd of a representative experiment (*n* = 40). Letters indicate statistically significant differences for each treatment at each time point (ANOVA, *P* < 0.05). Stomatal aperture ratio mean values ± Sd of a total of 40 stomata from 3 biological replicates of NT and α-terpineol-treated tomato. Stomatal aperture after α-terpineol treatment (blue points) and NT (gray points) ratio in **C)** ABA-deficient tomato mutants (*flacca*) and the corresponding parental (*Lukullus*) **D)***NahG* transgenic tomato plants impaired in SA accumulation and the corresponding parental MM. Data represent the mean ± Sd of a representative experiment (*n* = 50). Different letters indicate statistically significant differences for each treatment (ANOVA, *P* < 0.05). **E)** Bacterial content 24 h after infection in *NahG* tomato plants pretreated with 5 *µ*M α-terpineol or NT and then inoculated by immersion 1 d later. Data are presented as mean (log cfu/cm^2^) ± Sd of a representative experiment (*n* = 4). No statistically significant differences between treated and NT plants were observed after *t* test.

To better explore the function of α-terpineol in stomatal closure, weight loss was measured in tomato seedlings after water or α-terpineol treatments during 120 min (see Materials and methods). As shown in Fig. 2A, α-terpineol-treated plants statistically retained more water than nontreated plants, indicating an effective stomatal closure after the chemical treatment. We also compared the effect of α-terpineol with that produced by abscisic acid (ABA), the main phytohormone involved in stomata closure. We observed that treatments with α-terpineol, at a comparable range of concentrations as that used for ABA, can close stomata at a comparable range of concentrations as that used for ABA, although the observed effect was lower (Fig. 2B).

To test if the observed terpineol-induced stomatal closure is ABA or SA dependent, we measured the effect in tomato ABA-deficient *flacca* mutants (Bowman et al. 1984) and SA-deficient *NahG* transgenic plants (Brading et al. 2000). In addition to ABA, SA induces stomatal closure as a signaling molecule for plant defense responses to bacterial pathogens (Panchal and Melotto 2017), explaining the higher aperture ratio observed in nontreated *flacca* mutants and *NahG* transgenic plants. Terpineol treatments resulted in a significant stomatal closure in *flacca* and *NahG* (Fig. 2, C and D), thus indicating that the stomata closure effect of α-terpineol is SA and ABA independent.

To explore if the observed resistance upon α-terpineol treatment (Fig. 1C) was SA dependent, *NahG* tomato plants were pretreated with α-terpineol and then infected with *Pst*. As Fig. 2E shows, α-terpineol was unable to induce resistance in *NahG* tomato plants, thus indicating the effect of HMTPs on plant resistance is SA dependent.

### Alteration of *MTS1* gene expression levels affects stomatal closure but not *Pst* resistance

**Figure 3.**
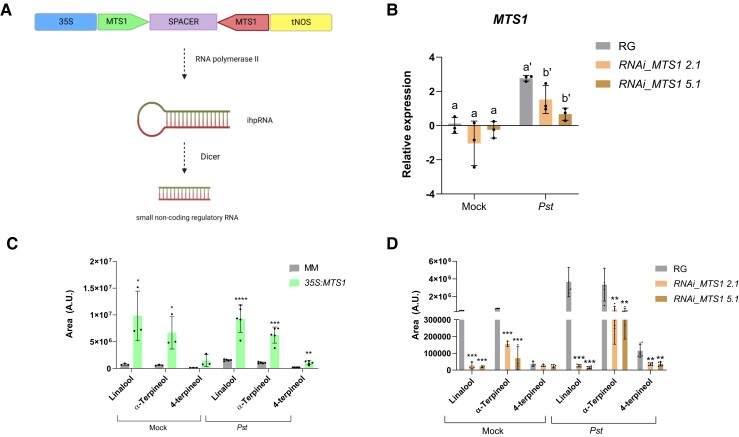
Characterization of transgenic plants with altered levels of monoterpenoids. **A)** DNA construction for the generation of transgenic plants *RNAi_MTS1*. **B)** Analysis of *MTS1* expression by RT-qPCR of the different *RNAi_MTS1* transgenic tomato lines (2.1 and 5.1) and its parental (RG) infected with bacterial (*Pst*) or noninoculated (Mock). RG and transgenic plants were subjected to infection with *Pst* by immersion. Samples were taken 24 h after the bacterial infection. The RT-qPCR values were normalized with the level of expression of Actin gene. The *y* axis represents the value of the Ct increment (ΔΔCt). The expression levels correspond to the mean ± Sd of a representative experiment (*n* = 3). Statistically significant differences (ANOVA, *P* < 0.05) between genotypes and *Pst*-infected or Mock plants are represented by different letters. Relative HMTP levels (arbitrary units [A.U.]) analyzed by GC–MS in tomato *35S:MTS1* leaves and their control transgenic plants with empty vector (MM; **C**) and lines of *RNAi_MTS1* 2.1 and 5.1 and their parental (RG; **D**) upon mock inoculation and *Pst* infection. Data are presented as means ± Sd of a representative experiment (*n* = 5). Statistically significant differences are represented with asterisk (*), double asterisk (**), triple asterisk (***) and quadruple asterisk (****) and indicate significant differences by *t* test with respect to genetic background (MM or RG) with *P* < 0.05, *P* < 0.01, *P* < 0.001 and *P* < 0.0001, respectively.

To provide genetic evidence confirming the observed defense-related effects triggered by HMTPs, tomato transgenic plants with altered levels of these volatiles were studied. Transgenic tomato plants overexpressing *MTS1* were previously described (van Schie et al. 2007), and silenced *MTS1* tomato plants were generated by following an RNAi strategy (see Materials and methods; Fig. 3A). Generated tomato line *RNAi_MTS1* was characterized, and homozygous lines *RNAi_MTS1 2.1* and *RNAi_MTS1 5.1*, both carrying 1 copy of the transgene, were selected for further studies. To characterize the response of *RNAi_MTS1* tomato plants to bacterial infection, the *MTS1* expression levels in mock-inoculated and *Pst*-infected plants were analyzed by RT-qPCR (Fig. 3B). As expected, a statistically significant reduction of *MTS1* transcript was measured in *Pst*-infected *RNAi_MTS1* leaves compared to corresponding infected wild-type RG.

To determine the emission of VOCs in both mock- and bacterial-infected *35S:MTS1* and *RNAi_MTS1* transgenic plants, a monoterpenoid targeted analysis was performed. Figure 3C shows that the emission of monoterpenoid-type VOCs was statistically higher in the *Pst*-infected *35S:MTS1* transgenic plants compared to MM wild-type plants. These results confirm the function described for *MTS1* as a monoterpene synthase and correspond with those previously described in which the basal levels of linalool reported in transgenic plants were higher than those in plants transformed with the empty vector (Van Schie et al. 2007). The chemical composition of *RNAi_MTS1* (Fig. 3D) also confirms that the silencing of *MTS1* upon a *Pst* infection causes a significant reduction of the HMTP emission, specifically linalool, terpinen-4-ol, and α-terpineol. Therefore, *MTS1* overexpression or silencing in infected tomato plants produced an opposite HMTP emission.

**Figure 4.**
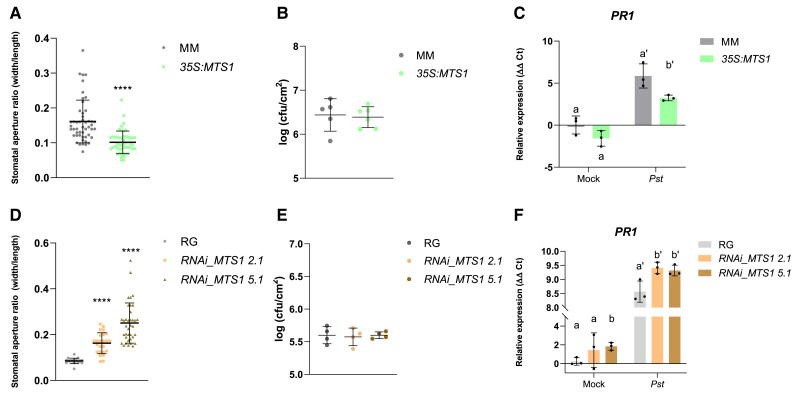
Activation of the defensive response in tomato plants with altered levels of monoterpenoids. Stomatal aperture, bacterial infectivity, and *PR1* gene expression were studied for transgenic tomato lines overexpressing **A to C)** or silencing *MTS1* gene **D to F)**. Stomatal aperture ratio mean values ± Sd of a total of 40 stomata from 3 biological replicates are shown in **A)**, for *35S:MTS1* leaves and their control transgenic plants with empty vector (MM), and in **D)**, for lines of *RNAi_MTS1* 2.1 and 5.1 and their parental (RG). Asterisks (****) indicate statistically significant differences between genotypes (*t* test, *P* < 0.0001). Growth of *Pst* are shown in leaves of **B)***35S:MTS1* plants and their parental (MM) and **E)** both silencing lines of *RNAi_MTS1* and their parental RG. Tomato plants were inoculated with bacterial *Pst* by immersion, and leaf samples were taken 24 h after bacterial infection. Data are presented as means (log cfu/cm^2^) ± Sd of a representative experiment (*n* = 5 and *n* = 4, respectively). RT-qPCR expression analysis of the tomato *PR1* gene are shown in **C)***35S:MTS1* plants and their parental (MM) and **F)** both silencing lines of *RNAi_MTS1* and their parental RG. Mock represents the noninoculated plants. The *y* axis represents the value of the Ct increment (ΔΔCt). Values were normalized to Actin gene. Expression levels are represented as mean ± Sd of 3 biological replicates of 1 representative experiment. Statistically significant differences (ANOVA, *P* < 0.05) between genotypes and infected (*Pst*) or mock-treated plants are represented by different letters.

To confirm the mode of action of HMTPs in the tomato defensive response, we checked the possible association between HMTP emission and stomatal closure. As shown in Fig. 4A, *35S:MTS1* transgenic plants displayed a lower ratio of stomatal aperture than the corresponding control plants. This result agrees with the stomatal closure observed with exogenous α-terpineol treatments (Fig. 1A), reaffirming the role of HMTPs in the regulation of stomatal closure. Also consistently, both transgenic lines silencing *MTS1*, with lower levels of HMTPs, displayed a higher aperture ratio than the wild-type RG plants (Fig. 4D). Thus, our results appear to indicate HMTPs can cause stomatal closure when provided exogenously but also when produced endogenously, probably being involved in stomatal immunity.

To test this possibility, a bacterial infection was carried out in both transgenic plants *35S:MTS1* and *RNAi_MTS1* as well as in their corresponding MM and RG parentals. Surprisingly, as seen in Fig. 4B, the *MTS1* overexpression line did not show enhanced resistance to *Pst*, despite having a lower stomata aperture ratio than their corresponding MM (Fig. 4A). Besides, *RNAi_MTS1* tomato plants did not display a higher susceptibility (Fig. 4E) irrespective of the previously observed higher stomata aperture ratio (Fig. 4D). These results are also in contrast with those obtained after exogenous treatments (Fig. 1C).

The expression levels of the pathogenesis marker *PR1* were measured in both infected transgenic plants. As shown in Fig. 4C, a statistically lower expression of *PR1* was detected in *35S:MTS1* transgenic plants after *Pst* infection when compared to that observed in MM wild-type plants, which is in contrast with the previously observed HMTP-mediated *PR1* induction (Fig. 1B). Contrarily, both *RNAi_MTS1* lines showed statistical higher levels of PR1 expression upon *Pst* infection (Fig. 4F).

The induction of *PR1* is SA dependent, being highly induced in tomato plants over accumulating this phenolic compound (Payá et al. 2022). In addition, we have observed that the effect of HMTPs on plant resistance is SA dependent (Fig. 2E). Therefore, the pattern of induction of *PR1* and the lack of resistance phenotype in the transgenic plants with altered HMTP production suggest that the SA pathway could be affected under stress conditions in these transgenic plants.

### Metabolic crosstalk between MEP and SA pathways in infected tomato plants

**Figure 5.**
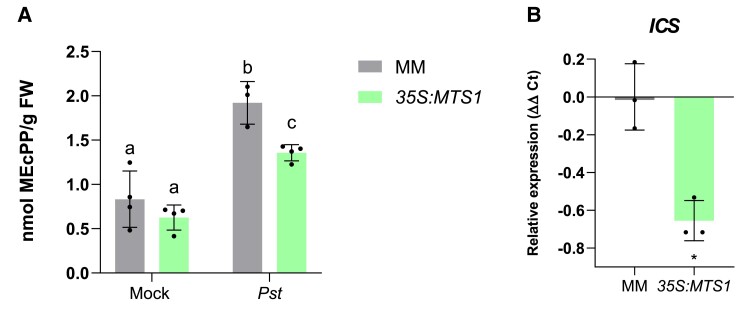
Reduction of MEcPP levels and *ICS* expression in *35S:MTS1* tomato transgenic plants. **A)** MEcPP content was measured (nmol/g FW, fresh weight) in overexpressing *35S:MTS1* plants and their control MM transgenic plants (*n* = 4) carrying an empty vector (MM) upon infection with *Pst*. Mock represents the noninoculated plants. Statistically significant differences (ANOVA, *P* < 0.05) between genotypes and infected (*Pst*) or mock-treated plants are represented by different letters. Levels are represented as mean ± Sd of 4 biological replicates. **B)***ICS* expression levels in infected *35S:MTS1* plants and their corresponding parentals with empty vector (MM; *n* = 3). The *y* axis represents the value of the Ct increment (ΔΔCt). Values were normalized to Actin gene. Expression levels are represented as mean ± Sd of 3 biological replicates of 1 representative experiment. Statistically significant difference (*t t*est, *P* < 0.05) between treated and nontreated is represented by an asterisk (*).

HMTPs are produced from precursors supplied by the plastidial MEP pathway. Interestingly, an intermediate of the MEP pathway, methylerythritol cyclodiphosphate (MEcPP), has been shown to transcriptionally activate *ICS*, which encodes the key enzyme of SA biosynthesis (Gil et al. 2005; Xiao et al. 2012). To study the possible competition for MEcPP to activate SA-mediated response or HMTP biosynthesis, measures of MEcPP levels were performed in *35S:MTS1* tomato plants upon *Pst* infection (Fig. 5A), observing a statistical reduction of this compound in *35S:MTS1* tomato plants. This reduction in MEcPP levels was accompanied by a significant reduction in the *ICS* expression in *35S:MTS1* infected tomato plants, when compared with the corresponding parental plants (Fig. 5B). The decrease in MEcPP levels and *ICS* expression caused by the overproduction of HMTPs (Fig. 3C) could be responsible for the observed lower levels of *PR1* (Fig. 4C) and the absence of resistance in *35S:MTS1* tomato plants (Fig. 4B).

To better study this interaction between HMTPs and SA biosynthesis, a pharmacological approach was followed to alter the levels of MEcPP, 24 h before *Pst* infection. Specifically, we used fosmidomycin (FSM) to block the early steps of the MEP pathway by inhibiting the enzyme deoxyxylulose 5-phosphate reductoisomerase (DXR) and reducing MEcPP production.

**Figure 6.**
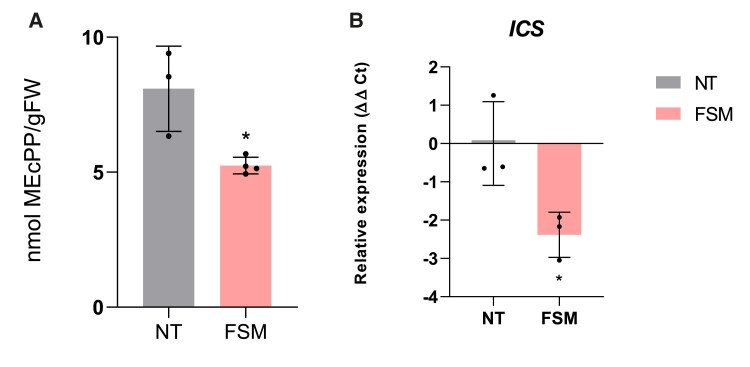
Reduction of MEcPP levels and *ICS* expression in MM tomato plants with alterations in the MEP pathway upon bacterial infection. **A)** MEcPP content after FSM treatment and their control nontreated (NT) plants (*n* = 4). The media levels of nmol MEcPP/g FW (fresh weight) ± Sd are represented, and statistically significant difference is represented by an asterisk (*; *t* test, *P* < 0.05). **B)***ICS* expression levels after FSM (*n* = 3) treatment and in NT plants. The *y* axis represents the value of the Ct increment (ΔΔCt). Values were normalized to *Actin* gene. Expression levels are represented as mean ± Sd of 3 biological replicates of 1 representative experiment. Statistically significant difference (*t* test, *P* < 0.05) between treated and NT is represented by an asterisk (*).

To confirm the inhibition of the MEP pathway, MEcPP levels were measured in *Pst*-infected tomato plants pretreated with FSM, observing a significant reduction of this compound in the pretreated leaves when compared with the corresponding nontreated plants (Fig. 6A). Then we studied the FSM inhibition effect of the MEP pathway on the *ICS* expression levels in *Pst*-infected tomato plants by RT-qPCR (Fig. 6B). Indeed, FSM application caused a statistical decrease in the *ICS* expression levels upon *Pst* infection, thus suggesting that the inhibition of the DXR enzyme blocked the MEcPP production and probably the transcriptional activation of *ICS*.

**Figure 7.**
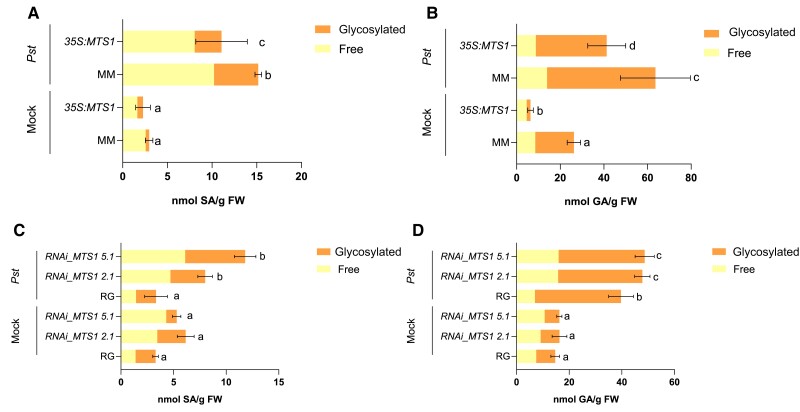
Metabolic crosstalk between monoterpenoids and SA biosynthesis. Levels of free and glycosylated salicylic (SA; left panels) and GA (right panels) were analyzed by fluorescence–HPLC in transgenic tomato plants with alterations in *MTS1* expression, 24 h after bacterial (*Pst*) infection. **A)** and **B)** show the phenolic content in overexpressing *35S:MTS1* tomato plants and their control plants with the empty vector (MM) and **C)** and **D)** in silencing lines of *RNAi_MTS1* tomato plants and their parental (RG). Mock represents the noninoculated plants. Bars represent the mean (nmol SA/g FW, fresh weight) ± Sd of total levels of a representative experiment (*n* = 4). Significant differences between genotypes and infected or mock-inoculated plants are represented by different letters (ANOVA, *P* < 0.05).

To confirm that regulation of *ICS* produces alterations in SA biosynthesis, we analyzed the levels of this phytohormone as well as its hydroxylated (GA) and methylated forms (MeSA), in both *Pst*-infected transgenic plants with altered *MTS1* levels. Both GA and MeSA are involved in compatible interactions and in the activation of SAR response (Lowe-Power et al. 2016), respectively. Particularly, MeSA has been proposed as the mobile signal responsible for the SAR activation in *Nicotiana tabacum* cv. Xanthi plants (Park et al. 2007). As shown in Fig. 7, A and B, the levels of SA and GA were statistically lower in *Pst*-infected *35S:MTS1* tomato leaves when compared to its genetic background MM. A significant reduction in GA levels was also measured in mock conditions in these transgenic plants. In contrast, a significant higher SA and GA accumulation was measured in *Pst*-infected *RNAi_MTS1* plants when compared with the corresponding parental plants (Fig. 7, C and D). Thus, the lower *ICS* induction observed in *MTS1* overexpressing lines (Fig. 5B) associated with the lower levels of SA and GA (Fig. 7, A and B) and with the lower expression of *PR1* upon bacterial infection (Fig. 4C). In a similar manner, an opposite MeSA emission was analyzed in *35S:MTS1* and *RNAi_MTS1* tomato plants. While *35S:MTS1* tomato leaves showed a lower emission of volatile MeSA after infection, *RNAi_MTS1* plants exhibited a higher production of MeSA compared to the corresponding wild type (Supplementary Fig. S2, A and D). Moreover, the expression levels of *S5H*, which is involved in the conversion of SA to GA (Payá et al. 2022), paired with the accumulation levels of these 2 phenolics in both infected transgenic plants. Thereby, *35S:MTS1* tomato leaves showed a statistical decrease in *S5H* expression levels while *RNAi_MTS1* displayed a slightly significant *S5H* induction after bacterial infection compared to the parentals (Supplementary Fig. S2, B and E). Finally, since MEcPP has also been shown (Xiao et al. 2012) to transcriptionally activate *HPL*, a hydroperoxide lyase participating in the biosynthesis of green leaf volatiles (GLVs) such as *Z*-3-hexenal, levels of this volatile were also measured in both transgenic plants, observing the same of accumulation pattern observed for SA (Supplementary Fig. S2, C and F).

**Figure 8.**
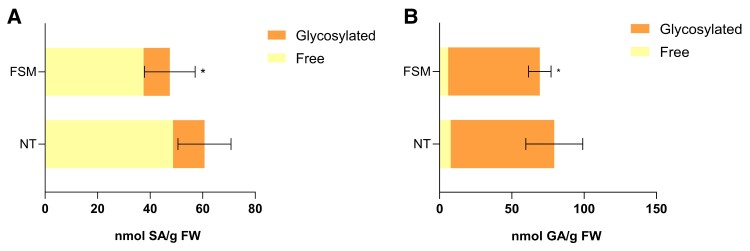
Pharmacological validation of the crosstalk between monoterpenoids and SA biosynthesis. Free and glycosylated **A)** SA (left panels) and **B)** GA (right panels) levels in FSM pretreated and nontreated (NT) infected MM tomato plants. The extracts were analyzed by fluorescence–HPLC. In both figures, bars represent the mean (nmol/g FW, fresh weight) ± Sd of total levels of a representative experiment (*n* = 4). Statistically significant differences (*t* test, *P* < 0.05) between treated and NT are represented by asterisks (*) of total levels of a representative experiment.

Furthermore, levels of SA and GA were also measured in tomato plants pretreated with FSM and infected with *Pst*, which displayed a lower activation of *ICS* (Fig. 6B). Also consistently, FSM-treated and infected tomato plants accumulated lower levels of SA (Fig. 8A) and GA (Fig. 8B), when compared with nontreated control plants infected with *Pst*. Once again, levels of accumulation of *Z*-3-hexenal associated with SA levels (Supplementary Fig. S3).

Both genetic and pharmacological approaches appear to indicate that there is a shared use of MEcPP by the MEP pathway, which leads to the production of HMTPs and the *ICS* transcriptional activation, which induces the SA biosynthesis, thus revealing a metabolic crosstalk between both pathways. These findings could explain the fact that, despite the observed statistical differences in HMTP emission and stomatal aperture ratios, none of the transgenic plants showed the expected phenotype of resistance (*35S:MTS1*) or susceptibility (*RNAi_MTS1*), since SA levels were inversely affected in these plants, thus confirming the existence of a negative crosstalk between HMTPs and SA during the bacterial infection.

### HMTPs and SA balance in the defensive response of tomato plants against *Pst*

**Figure 9.**
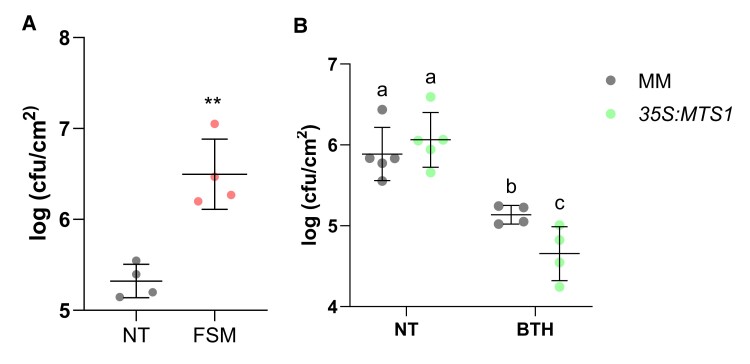
Role of the MEP pathway on the tomato resistance to bacteria. Tomato pretreated plants were inoculated with *Pst* by immersion, and leaf samples were taken 24 h after bacterial infection. Data are presented as means ± Sd of a representative experiment. Bacterial content in infected **A)** MM tomato plants nontreated (NT, *n* = 4) and pretreated with FSM (*n* = 4). Statistically significant differences (*t* test, *P* < 0.01) between treated and NT are represented by asterisks (**). **B)***35S:MTS1* transgenic tomato plants and their corresponding MM background with the empty vector (MM) NT (*n* = 5) and pretreated with BTH (*n* = 4). Data are presented as means (log cfu/cm^2^) ± Sd of a representative experiment. Statistically significant differences (ANOVA, *P* < 0.05) between genotypes and BTH-treated or NT plants are represented by different letters.

To verify the relevance of the MEP pathway and its connection with SA biosynthesis in plant resistance, FSM-pretreated tomato plants were infected with *Pst* and the bacterial growth was evaluated. As shown Fig. 9A, the number of colonies was statistically higher in FSM-treated plants with respect to the untreated ones. This result indicates that FSM treatments, which repressed *ICS* (Fig. 6B) and reduced SA accumulation (Fig. 8A), increase the susceptibility of tomato plants against *Pst*.

These treatments confirm the relation between the MEP pathway and SA biosynthesis and highlight the importance of HMTPs and SA in the plant resistance. These studies clarify the importance of the MEP pathway in the tomato resistance through MEcPP, a compound participating in both the transcriptional stimulation of *ICS* and the biosynthesis of HMTPs.

Finally, to confirm the role of HMTPs in plant defense against bacteria, treatments with the SA functional analog benzothiadiazole (BTH), used as chemical activator of plant resistance by activating SAR (Lawton et al. 1996), were carried out in the transgenic *35S:MTS1* tomato plants. Applications of 1 mM BTH in transgenic *35S:MTS1* plants rescued their SA deficiency, allowing HMTPs to produce a significant resistance when compared with corresponding treated parental, therefore mimicking HMTP treatments (Fig. 9B). This resistance was accompanied by a restored induction of *PR1* expression in the *35S:MTS1* plants (Supplementary Fig. S4) when compared with those previously obtained (Fig. 4C).

### HMTPs participate in the communication between tomato plants

**Figure 10.**
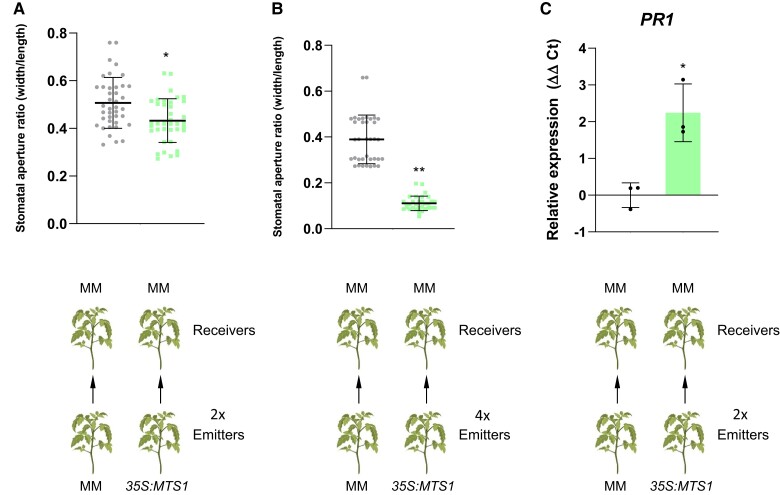
Interplant communication using *35S:MTS1* plants as emitters. Tomato MM plants (“Receivers”) were placed in closed chambers in the presence of *MTS1* overexpressing plants (“Emitters”) or their corresponding control plants with the empty vector MM as control Emitters, and stomatal aperture ratio of 3 biological replicates was measured in receivers of tomato plants after cohabitation for 24 h. Two emitters (2x) vs. 2 receivers were used in **A)**, and 4 emitters (4x) vs. 2 receivers were used in **B)**. The relative expression of tomato *PR1* gene **C)** was analyzed by RT-qPCR in MM receiver plants after cohabitation either with *35S:MTS1* or MM emitters. Two emitters vs. 2 receivers were used. The *y* axis represents the value of the Ct increment (ΔΔCt). Values were normalized to Actin gene. Expression levels are represented as mean ± Sd of 3 biological replicates of 1 representative experiment. Statistically significant differences (*t* test, *P* < 0.05 or *P* < 0.01) between treated and nontreated plants are represented by asterisks (*) or double asterisks (**), respectively.

The role of VOCs, in intra- and intercommunication between plants, is well known (Baldwin et al. 2002; Zimmermann et al. 2009). VOCs are long-distance signals that can trigger systemic stress responses in distant plants. As Fig. 10 shows, when tomato plants (receivers) were cohabited with *35S:MTS1* plants, which overemit HMTPs (emitters; Fig. 3C), stomatal closure was observed in those receiver plants. Moreover, the observed effect occurred in a dose-dependent manner, since the stomata closure was less pronounced when 2 emitter plants were used (Fig. 10A) in comparison with the results obtained with 4 emitter plants (Fig. 10B). Besides, *35S:MTS1* emitter plants activated the plant defense response in the receiver plants, as levels of *PR1* showed (Fig. 10C). Therefore, our results indicate that HMTPs may play an important role not only in the intra- but also in the interplant immune signaling.

## Discussion

Terpenoids constitute a highly diverse class of chemical compounds, which are abundantly produced across the plant kingdom (Zhou and Pichersky 2020). Particularly, monoterpenes are implicated in the plant defense response, being their defensive role classically associated with several plant–herbivore interactions (Sharma et al. 2017), i.e. *Arabidopsis thaliana*–*Myzus persicae* (Aharoni et al. 2003). However, its role in plant defense against pathogens is earning interest (Vlot et al. 2021). In this sense, some HMTPs as α-terpineol, 4-terpineol, or linalool have been described as differentially emitted VOCs during ETI establishment triggered by *Pst* in tomato plants (López-Gresa et al. 2017). Here, we have studied the defensive role of these HMTPs and its convergence with the SA-mediated immunity in tomato plants.

Stomata participate in the gas exchange that allows transpiration and photosynthesis, being involved in plant immunity as these apertures act as entry point for pathogens to the vegetal tissue (Melotto et al. 2008). The activation of plant defense leads to the accumulation of the so-called pathogenesis-related (PR) proteins, being PR1 the conventional SA-related marker (Saijo and Loo 2020). We have observed that all the analyzed HMTP treatments provoked both stomatal closure and *PR1* induction, unlike the nonhydroxylated limonene, thus pointing out the importance of the hydroxylation of monoterpenes for the activation of the plant defense response (Fig. 1, A to C). Interestingly, the α-terpineol-mediated disease resistance appeared to be due in part to its capability to close the stomata, since the resistance phenotype failed when bacteria were injected into the pretreated tomato plants (Fig. 1D). The importance of stomatal immunity in VOC-mediated resistance was also described by using (*Z*)-3-hexenyl butyrate, a volatile compound also emitted by tomato plants displaying ETI (López-Gresa et al. 2018).

To the best of our knowledge, HMTPs have not been reported as plant stomata closers. To date, previous research of Rai et al. (2003) indicated that volatile essential oils from *Prinsepia utilis* inhibit stomatal opening in *Vicia faba*. In particular, α-terpineol attracts a great interest as an antioxidant compound (Khaleel et al. 2018), being the most efficient volatile in terms of stomatal closure (Fig. 1A). To elucidate the defensive role of HMTPs, the capacity to activate plant resistance and the stomata closure process triggered by α-terpineol treatments were evaluated. This monoterpenoid induced *PR1* expression (Fig. 1B) and tomato resistance against *Pst* (Fig. 1C), confirming its defensive role. Our results are in accordance with those previously described in *A. thaliana*, where a mixture of the monoterpenes α-pinene and β-pinene induced resistance (Riedlmeier et al. 2017), although their capacity to induce stomatal closure was not explored. In other studies, monoterpenes had already been associated with the defensive response such as geraniol and its potential antibacterial effects against *Xanthomonas oryzae* pv. *oryzae*, which causes rice bacterial blight (Kiyama et al. 2021).

The effective stomata closure after α-terpineol treatment was confirmed by the reduced loss of water observed in treated plants (Fig. 2A), being the α-terpineol active at concentrations similar to those used for ABA (Fig. 2B), a plant hormone with a central role in the regulation of stomatal movements under water-deficit conditions (Hsu et al. 2021). Unexpectedly, the observed stomata closure occurred in a SA- and ABA-independent manner (Fig. 2, C and D), of these 2 positive regulators of the stomatal immunity (Melotto et al. 2006, 2017; Su et al. 2017). Similar results were previously described for oxylipins, which participate in the stomatal immunity in an ABA-independent process (Montillet et al. 2013), or for the (*Z*)-3-hexenyl butyrate, a volatile compound also emitted by tomato plants displaying ETI, which has been described to close stomata in a SA- and ABA-independent manner (Payá et al. 2003; López-Gresa et al. 2018). Our results reinforce the existence of ABA-independent pathways in stomatal immunity. Contrary to the stomata closure mode of action, we have observed that HMTPs induced resistance in a SA-dependent manner, since α-terpineol treatments were unable to induce resistance in *NahG* tomato plants (Fig. 2E). Similar results were previously described in Arabidopsis plants, where the SA-impaired mutants *sid2-1*, *eds1-2*, and *npr1-1* did not display enhanced resistance upon treatments with volatile pinenes, thus indicating that both SA signaling and biosynthesis are required for monoterpenoid-induced resistance (Riedlmeier et al. 2017).

In addition to the pharmacological approaches, the capacity of HMTPs to induce resistance was analyzed in transgenic plants with altered levels of *MTS1* expression. For this purpose, we used the previously described *35S:MTS1* plants (Van Schie et al. 2007), which overemit HMTPs, and we generated *RNAi_MTS1* plants with reduce emission levels of HMTPs (Fig. 3). Surprisingly, levels of emission of HMTPs did not correspond with plant resistance (Fig. 4, B and E), although the capacity of the HMTPs to regulate stomata was maintained in these transgenic plants (Fig. 4, A and D). Therefore, our results appeared to indicate the ability of HMTPs to confer resistance was not only due to stomatal closure. Expression levels of pathogenesis marker *PR1* were measured, observing a reverse association between HMTP emission and the expression of this defense marker gene (Fig. 4, C and F). These results suggested that HMTPs somehow alter *PR1* expression and, therefore, the SA-mediated response.

In Arabidopsis, there appears to be a relationship between monoterpenes and both SA biosynthesis and signaling. Specifically, pinene-induced resistance was described to be dependent of SA biosynthesis and signaling (Riedlmeier et al. 2017). In addition, *CSB3*, which encodes a 1-hydroxy-2-methyl-2-butenyl 4-diphosphate synthase participating in the MEP pathway, is expressed constitutively in healthy plants and shows repression in response to bacterial infection, being described as a point of metabolic convergence between MEP and SA-mediated disease resistance to biotrophic pathogens (Gil et al. 2005). Finally, the MEP pathway is connected to SA through *ICS* expression by the retrograde signaler MEcPP. This compound is a precursor of isoprenoids, which elicits the expression of selected stress-responsive nuclear-encoded plastidial proteins such as *ICS*, the main producer of SA (Xiao et al. 2012).

Accordingly, we observed that *35S:MTS1* transgenic plants displayed lower levels of MEcPP accumulation (Fig. 5A), a reduced induction of *ICS* (Fig. 5B), lower levels of SA (Fig. 7A), and therefore lowered PR1 activation (Fig. 4C) upon *Pst* infection. Conversely, higher levels of SA (Fig. 7C) and enhanced *PR1* expression (Fig. 4F) were detected in *RNAi_MTS1* plants. Unlike Arabidopsis and MM tomato plants, RG tomato plants did not display SA accumulation upon *Pst* infection. These results agree with those previously described (López-Gresa et al. 2017), thus revealing differences between tomato and Arabidopsis signaling defense.

SA levels in *35S:MTS1* and *RNAi_MTS1* could explain our observed phenotypes in tomato *MTS1* transgenic plants, since neither an enhanced resistance nor a susceptibility was obtained (Fig. 4, B and E), probably due to the alteration of the MEP pathway. In the case of *35S:MTS1* plants, the constitutive promoter would cause a depletion of MEP pathway precursors, which are routed into the biosynthesis of monoterpenes. Therefore, a lower amount of MEcPP could act as retrograde signal, reducing the *ICS* transcriptional activation. On the contrary, an accumulation of the MEP precursors, including MEcPP, occurs in *RNAi_MTS1* plants and *ICS* is then induced. The absence of phenotype in both cases account for the important role of both SA and HMTPs in tomato defense against bacteria.

To further confirm this last idea, we performed a pharmacological approach. Application of FSM, an inhibitor that targets the second enzyme of the MEP pathway reducing the levels of the final products (Laule et al. 2003; Di et al. 2022), was used to test the impact of eliminating both monoterpenoids and the transcriptional regulation of *ICS* in the defensive response. After FSM treatment, infected tomato plants displayed a downregulation of *ICS* (Fig. 6B), lower levels of SA (Fig. 8A), and consequently a higher susceptibility (Fig. 9A), confirming the connection between the MEP pathway and SA-mediated defense.

In conclusion, MEcPP is revealed as a key metabolite in tomato defense against biotic stress since it is necessary for monoterpenoid biosynthesis and positively regulates *ICS* expression. The lower levels of MEcPP detected in *35S:MTS1* confirmed this biosynthetic crosstalk (Fig. 5). In addition, the hypersusceptibility phenotype observed in tomato plants after treatments with FSM (Fig. 9A), as well as the enhanced resistance of *35S:MTS1* plants after BTH treatments against *Pst* (Fig. 9B), indicates that both HMTPs and SA are required for resistance induction in tomato plants. Furthermore, the biosynthetic regulation of both pathways mediated by MEcPP is key in the context of the defensive response and must be fine-tuned to optimize defense and fitness.

Finally, we have observed that *35S:MTS1* tomato plants induce stomata closure and *PR1* expression in neighbor receiving tomato plants, thus indicating that HMTPs play an important role in interplant communication (Fig. 10). Similar results were observed in Arabidopsis, since monoterpenes contributed to defense-related plant-to-plant communication (Riedlmeier et al. 2017). MeSA and nonanal are VOCs that have also been described to trigger plant defense (Shulaev et al. 1997; Yi et al. 2009). However, since *35S:MTS1* tomato plants emitted lower levels of MeSA (Supplementary Fig. S2A), the induction of plant defense in receiving plants is probably due to the HMTPs, reinforcing their role in the defense-related plant communication.

In summary, by using pharmacological and genetical approaches, we have demonstrated that HMTPs play an important defensive role in tomato, contributing to stomatal closure and the activation of the defense response not only within the plant but also in the neighboring plants, therefore acting as signal molecules for intra- and interplant communication. Moreover, our results have revealed a metabolic crosstalk between MEP and SA pathways, occurring through MEcPP competition.

## Materials and methods

### Vector construction and tomato transformation

In order to generate the *MTS1*-silenced transgenic tomato (*S. lycopersicum*) plants, the method described by Waterhouse and Helliwell (2003) was followed. Briefly, a selected 407 bp sequence of MTS1 was amplified from the full-length cDNA clone using the forward primer 5′-GGCTCGAGTCTAGAATGGTTTCAATATTGAGTAAC-3′, which introduced restriction sites *Xho*I and *Xba*I, and the reverse primer 5′-CCGAATTCGGATCCCTCCTCATAATTTGCATAATTTCATC-3′, which added restriction sites *Bam*HI and *Eco*RI. The PCR product was first cloned in the pGEM-T Easy Vector (Promega) and sequenced. After digestion with the appropriate restriction enzymes and purification, the 2 *MTS1* fragments were subcloned into the pHANNIBAL vector in both the sense and antisense orientations. Finally, the constructs in pHANNIBAL were subcloned as a *Not*I flanked fragment into a binary vector pART27 to produce highly effective intron-containing “hairpin” RNA-silencing constructs. This vector carries the neomycin phosphotransferase gene (*NPT II*) as a transgenic selectable marker.

The transformed LBA4404 *Agrobacterium tumefaciens* carrying pART27-MTS1 was cocultured with tomato RG cotyledons to generate the RNAi MTS1-silenced transgenic tomato plants (*RNAi_MTS1*). The explant preparation, selection, and regeneration methods followed those published by Ellul et al. (2003). The tomato transformants were selected in kanamycin-containing medium and propagated in soil. RG tomato wild-type plants regenerated in vitro from cotyledons under the same conditions as the transgenic lines were used as controls in subsequent analyses. The transgenic plants generated in this study have been identified and characterized in our laboratory and are to be used exclusively for research purposes.

### Plant material and growth conditions

For the purposes of this study, we used different tomato genotypes: (i) *NahG* (Brading et al. 2000) and its parental MM (kindly provided by Prof. Jonathan Jones, The Sainsbury Laboratory, Norwich, United Kingdom); (ii) *35S:MTS1* (Van Schie et al. 2007) and the parental MM with the empty vector pGreen (gently provided by Prof. Schuurink, Swammerdam Institute for Life Sciences, Department of Plant Physiology, University of Amsterdam, The Netherlands); (iii) *flacca* mutants and its parental Lukullus (Bowman et al. 1984; all of them kindly provided by Dr. Jorge Lozano, Instituto de Biología Molecular y Celular de Plantas UPV-CSIC, Valencia, Spain); and (iv) *RNAi_MTS1* plants generated in our laboratory and its parental RG that contain the *Pto* resistance gene (a gift from Dr. Selena Giménez, Centro Nacional de Biotecnología, Madrid, Spain). A mixture of sodium hypochlorite:distilled H_2_O (1:1) was used for the sterilization and sequential washings of 5, 10, and 15 min for the total removal of hypochlorite. Seeds germinated were placed in 12-cm-diameter pots with vermiculite and peat. The greenhouse conditions were the following: a relative humidity of 50 approximately and a 16/8-h (26 °C) light/dark photoperiod.

### HMTP treatments and interplant communication

Treatments were carried out in 4-wk-old tomato plants. Tomato plants were placed into 121-L methacrylate chambers containing hydrophilic cotton buds soaked with 5 *μ*M monoterpenoid in 0.05% (v/v) Tween-20 or distilled water. Methacrylate chambers were hermetically sealed during 24 h. For spray treatments, tomato plants were pretreated by spray with 2 mM monoterpenoid with 0.05% (v/v) Tween-20 or distilled water.

For interplant communication assays, 2 or 4 emitter plants were cohabitated with 2 receiver plants in the mentioned methacrylate chambers for 24 h. Plant material was only collected from the MM wild-type receiver plants.

### Inhibitors and BTH treatments

Twenty-eight-day-old MM plants were sprayed with 50 *µ*M FSM in 0.05% (v/v) Silwet detergent solution. One millimolar of BTH was applied 24 h before the infection with 0.05% (v/v) Silwet detergent solution.

### Bacteria inoculation and cfu determination

The bacterial strain used in this study was *P. syringae* pv. *tomato* DC3000 with deletions in genes *avrPto* and *avrPtoB* (*Pst*; Lin and Martin 2005; Ntoukakis et al. 2009). Bacterial growth conditions and plant inoculation were performed as previously described (López-Gresa et al. 2018). Briefly, bacterial inoculation was carried out in 4-wk-old tomato plants by immersion or infiltration. Tomato plants were dipped into the bacterial suspension with an optical density of 0.1 at 600 nm containing 0.05% Silwet L-77. To carry out bacterial infiltration experiments, each leaflet of the 3rd and 4th leaves was inoculated with a needleless syringe by pushing the bacterial suspension into different sites of the leaflet's abaxial side.

Briefly, for cfu measurements, 3 leaf disks (1 cm^2^ each) were grounded and serial dilutions of the infected tissue were cultured on King's B agar medium Petri dishes containing rifampicin. cfu were counted after incubation at 48 h at 28°C.

### Stomatal aperture

For the observation of aperture ratio, stomatal samples were imprinted with a layer of nail polish in the abaxial part of the leaves, and the epidermis peels were placed on slides for their observation with a Leica DC5000 microscope (Leica Microsystems S.L.U.). In total, 50 stomata of each condition were analyzed using the NIH's ImageJ software. Several pictures were taken from different regions of the tomato leaves. Stomatal aperture ratio was calculated as width/length.

### Weight loss experiments

Around 15 to 25 tomato plants were germinated in watered paper in Petri dishes in an in vitro chamber for 10 d, and then they were transferred to a MS Petri dish containing a sterile piece of hydrophilic cotton with enough α-terpineol to give us a final concentration of 5 *µ*M α-terpineol, considering the whole volume of the dish. Plants were maintained for 24 h in this treatment and then transferred outside the dish to an empty opened dish at room temperature that was left in a precision balance scale. The weight loss was monitored and registered every minute for 3 h, and then a final measure was performed after 24 h in order to leave the plants dry out completely.

### RNA isolation and RT-qPCR analysis

The RNA extraction and conversion to cDNA of tomato leaves were carried out using column kit based on silica membranes (Macherey-Nagel GmbH, Germany) following the manufacturer's protocol. cDNA from a microgram of RNA was obtained using a PrimeScript RT reagent kit (Perfect Real Time, Takara Bio Inc., Otsu, Shiga, Japan) following its instructions. qPCRs were performed as previously described (Campos et al. 2014). In each plate of a 96-well plate, a reaction took place in a final volume of 10 *µ*L. SYBR R Green PCR Master Mix (Applied Biosystems) was used as the fluorescence marker and actin gene as the endogenous reference gene. The RT-qPCR primers were designed using the online service Primer3 (http://primer3.ut.ee/) and are listed in Supplementary Table S1.

### SA and GA measurements

For the extraction of SA and GA, 0.5 g of frozen homogenized leaves was resuspended in methanol that contained 25 mM *o*-anisic acid as internal standard. The supernatant, after a 10-min centrifugation and 10-min sonication, was divided into 2 Eppendorf tubes and dried using a nitrogen flow. For the analysis of total and glycosylated SA and GA, the protocol described by Vázquez Prol et al. (2021) was carried out. Quantification of SA and GA was obtained using a calibration curve based on the internal standard.

### GC–MS

For the analysis of VOCs, a mix of 1 mL of CaCl_2_ 6 M and 100 *µ*L of 750 mM EDTA at a pH of 7.5 was added to 100 mg of pulverize tomato leaves in a 10-mL glass vial. The vials were airtight sealed and sonicated for 5 min. Volatile compound extraction was performed by head-space solid-phase microextraction (HS-SPME; López-Gresa et al. 2017). Enhanced ChemStation software (Agilent) was the program used to obtain and analyze the chromatograms and mass spectra, which has its own database to compare the different ion and retention times with pure compounds. Quantification of monoterpenes was performed elaborating a method in Agilent using the most abundant ion and retention time and calculating the area on the chromatogram. For VOC quantification, the following *m/z* quantifier ions and retention times were used: (i) α-terpineol: 93/121 and 30.6 min; (ii) linalool: 55/43 and 27.02 min; and (iii) 4-terpineol: 93/111 and 30.2 min.

### MEcPP measurements

MEcPP was quantified according to the protocol described in Baidoo et al. (2014) with minor modifications. One hundred milligrams of frozen homogenized tissue was extracted in 13 mM ammonium acetate buffer (pH 5.5). The extract was dried under a nitrogen steam and resuspended in the UPLC mobile phase (73% acetonitrile/27% 50 mM ammonium carbonate in water [v/v]).

MEcPP quantification was performed using a Orbitrap Exploris 120 mass spectrometer coupled with a Vanquish UHPLC System (Thermo Fisher Scientific, Waltham, MA, United States). LC was carried out by reverse-phase ultraperformance liquid chromatography using a BEH Amide column (1.7 *µ*m particle size, dimensions 2.1 × 150 mm; Waters Corp.)

Samples were run in isocratic mode for 14 min. The flow rate was 0.2 mL/min, and the injection volume was 5 *µ*L. The column temperature was set at 30°C.

Ionization was performed with heated electrospray ionization (H-ESI) in positive mode. Samples were acquired in full scan mode (resolution set at 120,000 measured at full width at half maximum). Methionine sulfone and D4-succinic acid were used as internal standards. For absolute quantification, a calibration curve was performed with MEcPP chemical standard. Data processing was performed with TraceFinder software (Thermo Scientific, Waltham, MA, United States).

### Western blot

Protein extracts for immunodetection experiments were prepared from MM plants treated and nontreated with α-terpineol for 24 h. Material (100 mg) for direct Western blot analysis was extracted in Laemmli buffer (125 mM Tris-HCl, pH 6.8, 4% [w/v] SDS, 20% [v/v] glycerol, 2% [v/v] 2-mercaptoethanol, and 0.001% [w/v] bromophenol blue), and proteins were run on a 14% SDS–PAGE gel and analyzed by immunoblotting. Proteins were transferred onto Immobilon-P membranes (Millipore) and probed with antirabbit peroxidase (Jacksons). Immunodetection of defensive protein PR1 was performed using antiPR1 antibody. Antibodies were used at a 1:20,000 dilution. Detection was performed using the ECL Advance Western Blotting Chemiluminiscent Detection Kit (GE Healthcare). Image capture was done using the image analyzer LAS3000, and quantification of the protein signal was done using Image Gauge V4.0 software.

### Statistical analysis

The statistical analysis of 2 or more variables was carried out using Student's *t* test or analysis of variance, respectively, employing GraphPad Prism9 software. For *t* test analyses, *P* < 0.05, *P* < 0.01, *P* < 0.001, and *P* < 0.0001 correspond to *, **, ***, and ****, respectively. For ANOVA analyses, a *P* < 0.05 was considered statistically significant and indicated with different letters.

### Accession numbers

Sequence data from this article can be found in the GenBank/EMBL data libraries under the following accession numbers: Y08804 (*PR1b1*), Solyc03g080190 (*S5H*), AY840091 (*MTS1*), and ICS (Solyc06g071030).

## Supplementary Material

kiae148_Supplementary_Data

## Data Availability

All data are incorporated into the article and its online supplementary material.

## Supplementary data

The following materials are available in the online version of this article.

**Supplementary Figure S1**. Western blot analysis of PR1 (14 kDa) accumulation in tomato leaves after 24 h of α-terpineol treatments.

**Supplementary Figure S2**. Changes in the MeSA content, the relative expression levels of the tomato *S5H* gene, and the *Z*-3-hexenal content in plants with alterations in *MTS1* upon bacterial infection.

**Supplementary Figure S3**. Changes in the *Z*-3-hexenal content in plants treated with inhibitors of the of the MEP pathway.

**Supplementary Figure S4**. Effect of BTH treatments in the resistance of *35S:MTS1* transgenic plants.

**Supplementary Table S1**. Primer sequences used for RT-qPCR analyses.

## Dive Curated Terms

The following phenotypic, genotypic, and functional terms are of significance to the work described in this paper:

DXR Gramene: AT5G62790
DXR Araport: AT5G62790

SDS Gramene: AT1G14750
SDS Araport: AT1G14750

EPS1 Gramene: AT5G67160
EPS1 Araport: AT5G67160

PR1 Gramene: AT2G14610
PR1 Araport: AT2G14610

ETI Gramene: Effector-triggered immunity
ETI Araport: Effector-triggered immunity
