# Supplementary material for: Metabolic crosstalk between hydroxylated monoterpenes and salicylic acid in tomato defense response against bacteria
Source: Plant Physiol. 2024 Mar 13;195(3):2323–38. doi: 10.1093/plphys/kiae148 (PMC11213251; doi:10.1093/plphys/kiae148)
Supplement: kiae148_Supplementary_Data [file kiae148_supplementary_data.pdf]

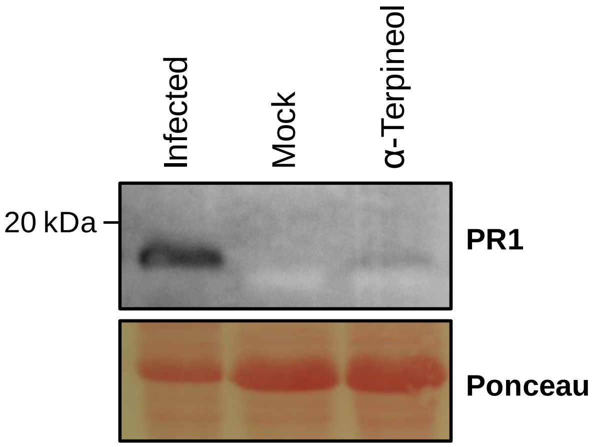

Supplemental Figure S1. Western blot analysis of PR1 (14 kDa) accumulation in tomato leaves after 24 h of α-terpineol treatments.

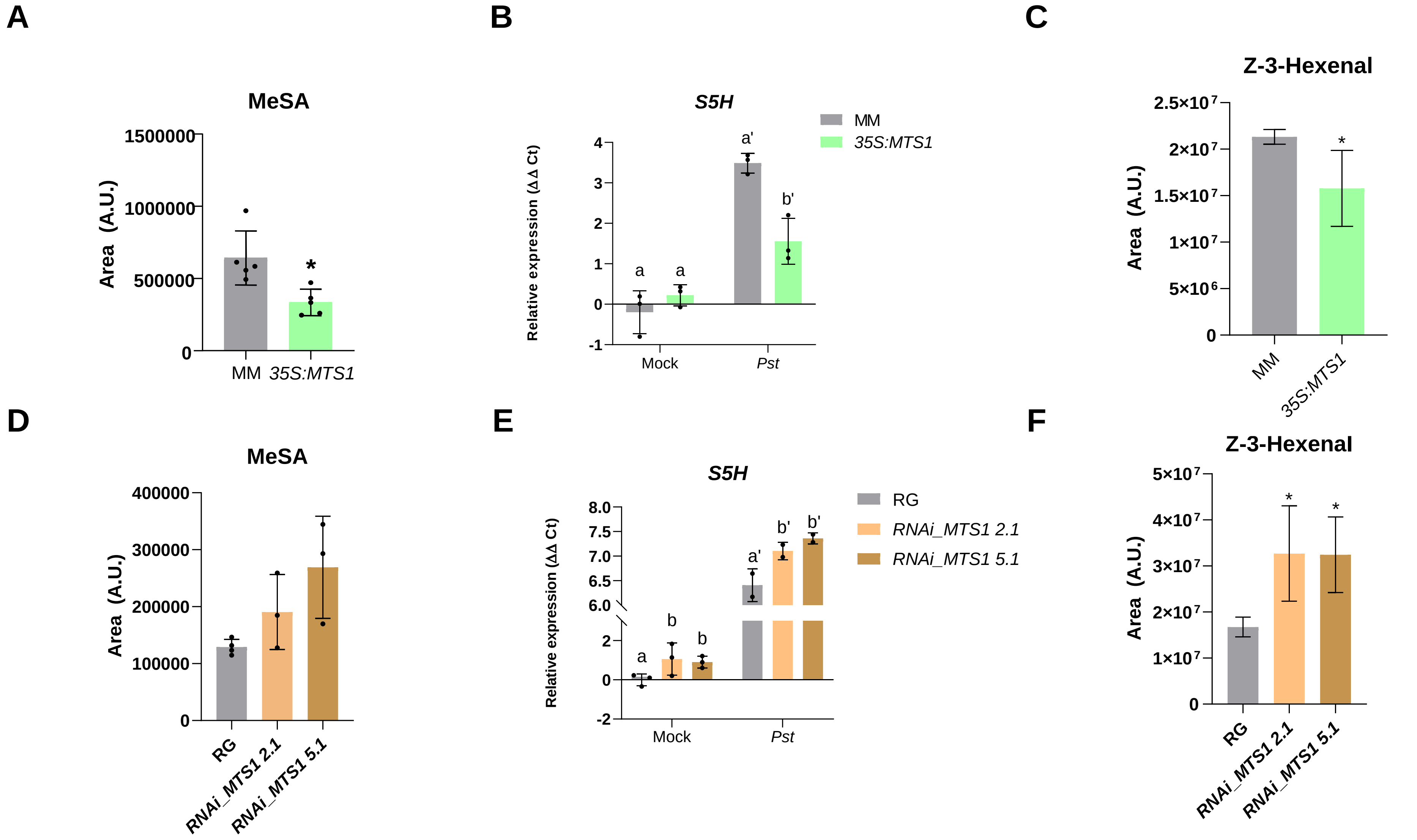

Supplemental Figure S2. Changes in the methyl salicylate (MeSA) content, the relative expression levels of the tomato *S5H* gene and the *Z*-3-Hexenal content in plants with alterations in *MTS1* upon bacterial infection. Upper panels. Data corresponding to *MTS1* overexpressing plants and their parentals carrying an empty vector (MM). **Lower panels.** Data corresponding to silencing lines of *RNAi_MTS1* (2.1 and 5.1) and their parental (RG). Relative levels (A.U. Arbitrary Units) of MeSA (**A** and **D**) and Z-3-hexenal (**C** and **F**). Statistically significant differences (*t*-test) with non-transgenic plants are represented with asterisk (*) with $p < 0.05$ (at least n=3). Data represents means ± SD (standard deviation). The RT-qPCR expression analysis of the tomato *S5H* gene is shown in **B)** and **E).** The y axis represents the value of the Ct increment (ΔΔCt). Expression values were normalized to Actin gene. Expression levels are represented as mean ± SD of three biological replicates of one representative experiment. Letters represent statistically significant differences (ANOVA, $p < 0.05$) between genotypes and infected (*Pst*) or mock-treated (Mock) plants.

A

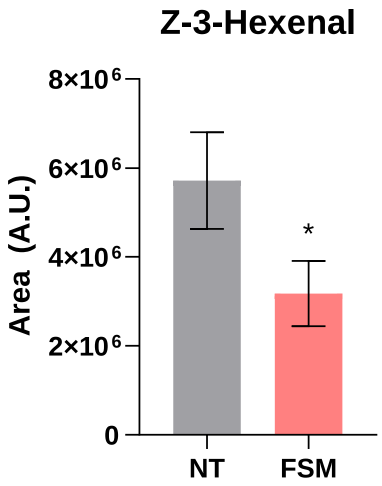

Supplemental Figure S3. Changes in the *Z*-3-Hexenal content in plants treated with inhibitors of the of the MEP pathway. Relative levels of *Z*-3-Hexenal in fosmidomycin (FSM) pre-treated and non-treated (NT) infected MoneyMaker tomato plants. Bars represent the mean (A.U. Arbitrary units) ± SD of total levels of a representative experiment (n=4). Statistically significant differences (*t*-test, p < 0.05) between treated and non-treated are represented by an asterisk (*).

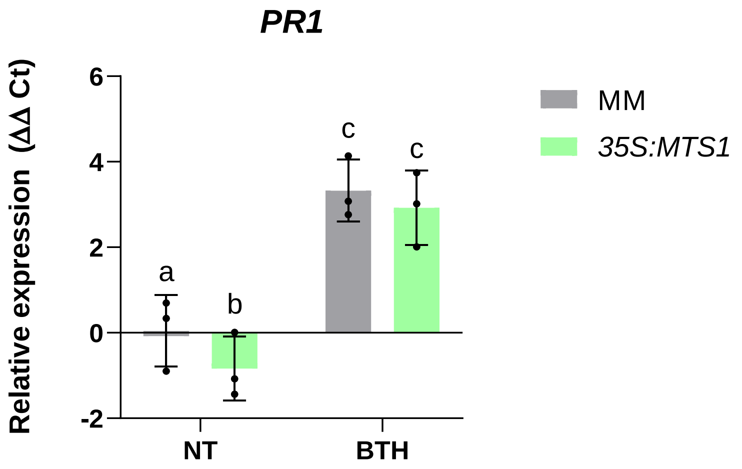

Supplemental Figure S4. Effect of benzothiadiazole (BTH) treatments in the resistance of *35S:MTS1* transgenic plants. Relative expression analysis by RT-qPCR of the tomato *PR1* gene in *35S:MTS1* plants and their control transgenic plants with empty vector (MM) after BTH and water (NT; non-treated) treatments. The y axis represents the value of the Ct increment (ΔΔCt). Values were normalized to Actin gene. Expression levels are represented as mean ± SD of three biological replicates of one representative experiment. Statistically significant differences (ANOVA, $p < 0.05$) between genotypes and treated plants are represented by different letters.

Gen	*Foward Primer*	*Reverse Primer*
ICS	**5’ TGC CTC ATG GAC ATA CCA GA 3’**	**5’ TAT GCG AAT GGG GAT TTT TTC 3’**
PR1	**5’ ACT CAA GTA GTC TGG CGC AAC TCA 3’**	**5’ AGT AAG GAC GTT GTC CGA TCG AGT 3’**
S5H	**5’ GGG ATG TCC CGG AAG TAA GT 3’**	**5’ GGC ATT GGA TGG GAT ATT CA 3’**
MTS1	**5’ TGG TGG TCA CCT TCA AGA GA 3’**	**5’ GCC TTG TGG AAA TAG GA 3’**
Actin	**5’ CTA GGC TGG GTT CGC AGG AGA TGA TGC 3’**	**5’ GTC TTT TTG ACC CAT ACC CAC CAT CAC AC 3’**

Supplemental Table S1. Primer sequences used for RT-qPCR analyses.
